# Evaluation of Two Commercially Available DNA Tests for Detection of Human Papillomavirus

**DOI:** 10.1155/S1064744995000147

**Published:** 1995

**Authors:** Diane C. Halstead, Sharon L. H. Pfleger, William Dupree

**Affiliations:** 1 Microbiology/Virology Division Lehigh Valley Hospital Allentown PA USA; 2 Pathology Department Lehigh Valley Hospital Allentown PA USA; 3 Microbiology Division Warren Hospital Phillipsburg NJ USA; 4 Baptist Medical Center Microbiology 3N 800 Prudential Drive Jacksonville FL 32207 USA

## Abstract

*Objective:* This study was designed to compare the sensitivity, specificity, efficiency, positive and negative predictive values, and ease of use for 2 commercially available hybridization kits for detecting human papillomavirus (HPV) DNA: Oncor Southern blot (SB) (Oncor, Inc., Gaithersburg, MD) and Digene ViraType dot blot (DB) (Digene Diagnostics, Inc., Silver Spring, MD).

*Methods:* A total of 179 specimens (172 cervical and 7 penile biopsies) were assessed for acceptability based on the presence of epithelial cells and tested for HPV by DB and SB. The results were evaluated based on Papanicolaou-stained cervical specimens and selected risk factors.

*Results:* One hundred six (97.2%) of 109 results were concordant, i.e., 93 negative (85.3%) and 13 positive (11.9%). Using SB as the gold standard, we found the sensitivity, specificity, efficiency, and positive and negative predictive values for the ViraType DB to be 100%, 96.9%, 97.3%, 81.3%, and 100%, respectively. Comparing the Papanicolaou smear to SB and DB, we found the sensitivity, specificity, efficiency, and positive and negative predictive values to be 33.3% (SB) vs. 44.4% (DB), 89.5% vs. 87.6%, 87.3% vs. 84.2%, 11.8% vs. 23.5%, and 97.0% vs. 94.9%, respectively. The only significant risk factor for predicting an HPV infection was the number of sexual partners.

*Conclusions:* Although SB has been considered the standard model, DB is an acceptable method for detecting and identifying HPV infections.


Infectious Diseases in Obstetrics and Gynecology 2:255–262 (1995)
© 1995 Wiley-Liss, Inc.

### Evaluation of Two Commercially Available DNA Tests for Detection of Human Papillomavirus

**Diane C. Halstead, Sharon L.H. Pfleger, and William Dupree**

*Microbiology/Virology Division (D.C.H.), Pathology Department (W.D.), Lehigh Valley Hospital, Allentown, PA, and Microbiology Division (S.L.H.P.), Warren Hospital, Phillipsburg, NJ*

### Abstract

***Objective:*** **This study was designed to compare the sensitivity, specificity, efficiency, positive and negative predictive values, and ease of use for 2 commercially available hybridization kits for detecting human papillomavirus (HPV) DNA: Oncor Southern blot (SB) (Oncor, Inc., Gaithersburg, MD) and Digene ViraType dot blot (DB) (Digene Diagnostics, Inc., Silver Spring, MD).**

***Methods:*** **A total of 179 specimens (172 cervical and 7 penile biopsies) were assessed for acceptability based on the presence of epithelial cells and tested for HPV by DB and SB. The results were evaluated based on Papanicolaou-stained cervical specimens and selected risk factors.**

***Results:*** **One hundred six (97.2%) of 109 results were concordant, i.e., 93 negative (85.3%) and 13 positive (11.9%). Using SB as the gold standard, we found the sensitivity, specificity, efficiency, and positive and negative predictive values for the ViraType DB to be 100%, 96.9%, 97.3%, 81.3%, and 100%, respectively. Comparing the Papanicolaou smear to SB and DB, we found the sensitivity, specificity, efficiency, and positive and negative predictive values to be 33.3% (SB) vs. 44.4% (DB), 89.5% vs. 87.6%, 87.3% vs. 84.2%, 11.8% vs. 23.5%, and 97.0% vs. 94.9%, respectively. The only significant risk factor for predicting an HPV infection was the number of sexual partners.**

***Conclusions:*** **Although SB has been considered the standard model, DB is an acceptable method for detecting and identifying HPV infections.** © 1995 Wiley-Liss, Inc.

Key words

Papillomavirus DNA, hybridization kits, HPV infection, Papanicolaou smear, SIL

During the last 10 years, there have been major changes in the methods used to diagnose and manage cervical squamous-cell cancer and its precursors.[1–3] More recently, molecular, clinicopathologic, viral, and DNA studies have continued to demonstrate similarities between condyloma and cervical intraepithelial neoplasia (CIN) I and between CIN II and CIN III.[4] These similarities are reflected in the Bethesda System for reporting Papanicolaou-stained smears which divides the spectrum of morphologic squamous changes into low-grade squamous intraepithelial lesion (SIL) (condyloma, CIN I) and high-grade SIL (CIN II, CIN III).[5] There is almost universal agreement that untreated patients with high-grade SIL, especially those with the equivalent of CIN III, are at significantly elevated risk of developing invasive squamous-cell carcinoma over time.

The natural history of low-grade SIL is much more controversial, although a consensus is emerging that most low-grade SILs have a high spontaneous regression rate and are unlikely to progress to invasive cancer.[6] Despite this generally favorable view of low-grade SIL, many published prospective studies document cases of low-grade SIL that have seemingly evolved into high-grade lesions.[7,8]

Address correspondence/reprint requests to Dr. Diane C. Halstead, Baptist Medical Center, Microbiology 3N, 800 Prudential Drive, Jacksonville, FL 32207.

Received August 10, 1994
Accepted January 30, 1995

Clinical Study

Further confounding the issue is the fact that statements in regard to the natural history of SILs are based on population statistics. Hence, the individual patient and her physician are faced with a probability statement as to what her unique course might be. What is needed is a means to determine which individual low-grade SILs will progress or persist and which will regress.

Although unequivocal evidence establishing that human papillomavirus (HPV) alone is the cause of cervical cancer and its precursor lesions is lacking, the strong association between HPV infection and these disease processes spurs intense efforts to better define the pathogenetic role of HPV.[6,9] Of >70 different types of HPV that infect humans, >20 have a predilection for the genital tract.[10] These can be divided into 3 groups: those with little or no oncogenic risk, those with an intermediate oncogenic risk, and those with a high oncogenic risk.[11] Based on a stratification of HPV types into oncogenic risk groups, Reid and Lorincz[12] have suggested that determining which patients with low-grade SIL will require treatment and which may be followed should be based in part on the use of HPV typing. These authors suggested that the patients whose lesions were associated with low-oncogenic-risk viral types might require neither therapy nor close clinical follow-up, whereas those lesions associated with high-oncogenic-risk HPV types should be treated "appropriately." Lesions with intermediate oncogenic viral types could be closely followed prospectively. Apart from the potential benefits for the patient, in an era of medical cost containment, this type of triage protocol has great appeal.

One of the problems associated with this triage strategy has centered around the reported insensitivity of commercially available HPV typing.[13] This study was designed to compare the sensitivity, specificity, efficiency, positive and negative predictive values, and the ease of use for 2 commercially available hybridization kits: Oncor Southern blot (SB) (Oncor, Inc., Gaithersburg, MD) and Digene ViraType dot blot (DB) (Digene Diagnostics, Inc., Silver Spring, MD). These kits utilize biotinylated and $^{32}P$-labeled RNA probes, respectively. The results from these 2 methods were correlated with cytology findings and patient risk factors.

### SUBJECTS AND METHODS

### Specimens

A double-blind coded study was performed on a total of 179 genital specimens collected between May 1991 and February 1992. There were 172 cervical specimens, including 146 from a private physician's office and 26 from the Lehigh Valley Hospital OB/GYN clinic, and 7 penile biopsies from condylomatous lesions collected in an ambulatory surgical unit. The women were asked to sign consent forms and to complete questionnaires in order to assess their risk factors and clinical histories. The following information was obtained from the questionnaire: age at first sexual encounter, number of sexual partners, use of barrier protection, previous sexually transmitted disease(s), abnormal Papanicolaou smear(s), previous gynecological infections, family history of neoplastic disease, and compromised immune system.

### Specimen Collection and Processing

Exfoliated cervical cells were collected using a sterile Christmas-tree cytobrush (Cat. #CYB, Medical Packaging Corp., Camarillo, CA). Three sequential cervical specimens were collected under direct visualization. A smear for Papanicolaou staining was prepared from the first cervical specimen. The remaining 2 specimens were placed in Digene transport medium (Digene Diagnostics, Inc.) and refrigerated for not more than 2 weeks, coded, evaluated for adequacy, and frozen at −70°C before processing. The biopsy specimens were hand-delivered to the laboratory in sterile tubes, coded, and frozen in phosphate-buffered saline at −70°C before processing.

The Papanicolaou smears were reviewed by a member of the cytology staff and a cytopathologist in accordance with the Bethesda System of nomenclature. The results obtained from the smears were placed into 7 categories for data analysis as follows: 1) normal; 2) infection, not HPV, bacteria/fungi; 3) inflammation with associated cell changes; 4) squamous atypia of undetermined significance, suggest repeat; 5) SIL-L (CIN I), condylomas, hyperkeratosis or parakeratosis suggestive of HPV, or koilocytes; 6) SIL-H (CIN II, CIN III), carcinoma in situ (CIS); and 7) unacceptable. The previous cytology results were also noted.

Each pair of specimens in transport medium was vortexed prior to removal of the cytobrushes, combined, and tested for adequacy by counting the number of squamous and columnar epithelial cells/low-power microscope field (lpf) in a wet-mount preparation. A specimen was considered adequate if at least an occasional cell (≥1 cell/lpf) was observed. Using the established criteria, we found 167/172 (97.1%) cervical specimens to be acceptable. The results were not available for 3/5 remaining specimens; 2 specimens were unacceptable. The presence of blood was also noted. The combined specimens were evenly distributed between 2 tubes (approximately 1 ml each) and frozen at −70°C until tested.

### Oncor SB Procedure

The specimens were tested according to the manufacturer's directions (Oncor, Inc.) using the Oncor Probe Tech automated electrophoresis instrument. The Oncor kit included "antisense" biotinylated probes with nucleic-acid sequences complementary to the entire viral genome of HPV types 6, 11, 16, 31, 33, and 35 and E6/E7 early open-reading frame (ORF) and L1 late ORF of HPV 18.

The results were interpreted according to the criteria established by Oncor. High and low concentration controls were used to identify the HPV-type specific bands. The procedure allows the identification of types 6, 11, 16, 18, 31, 33, and 35. If a sample did not form a band consistent with one of these HPV types, it was recorded as an "other" HPV type.

### Digene ViraType DB Procedure

The ViraType kit was obtained from Digene Diagnostics, Inc. The kit includes $^{32}$P-labeled RNA probes for the detection of HPV groups 6/11, 16/18, and 31/33/35. The probes are prepared by in vitro transcription of recombinant plasmid containing nearly the entire DNA sequences of HPV. With the exception of 2 specimens, visibly bloody specimens were not tested, according to the manufacturer's recommendations.

To determine the HPV type in a specimen, we compared the autoradiographic signal that was generated by the specimen with the positive control for the corresponding HPV probe group. Any signal greater than the negative control was considered positive. To distinguish between infection with a single type and one with multiple types, we compared the signals obtained with each positive patient blot and control. If the test specimen gave an autoradiographic signal with only one probe, the specimen was scored positive for that group of HPV types, e.g., 16/18. If the same specimen also exhibited a signal with another probe, e.g., 31/33/35, which was less than or equal to the signal produced by the 16/18 positive control on the membrane with the 31/33/35 probe, the specimen was scored negative for 31/33/35. If the test specimen gave a positive signal that was greater than the 16/18 positive control, a multiple infection could not be ruled out.

### Statistical Analysis

The statistical significance of the SB and DB results was determined using Youden's square. Contingency tables were used to analyze the risk factors and cytology results.

### RESULTS

### Correlation of SB and DB

One hundred seventy-nine specimens (172 cervical specimens from females between the ages of 13 and 77 years and 7 penile biopsies) were collected for HPV typing. A total of 109 specimens (102 cervical scrapings and 7 penile biopsies) were tested by both SB and DB. Seventy additional specimens were tested by either SB (54) or DB (16). Fifty-four of the 172 cervical specimens were visibly bloody and were not tested by DB because, according to the manufacturer's directions, bloody specimens may produce false negative results. Sixteen additional specimens were only tested by ViraType.

Sixteen (14.7%) of the specimens (9 cervical and all 7 biopsies) were positive by either SB or DB. Complete concordance between SB and DB assays was obtained with 106/109 (97.3%) specimens: 93/106 (87.7%) negative and 13/106 (12.3%) positive results. Three discordant specimens were positive by DB and negative by SB (Fig. 1). Using SB as the gold standard, we found the DB method to have sensitivity, specificity, efficiency, and positive and negative predictive values of 100%, 96.9%, 97.3%, 81.3%, and 100%, respectively ($P$ = 0.000). All 7 penile biopsies were positive by both methods. Table 1 lists the HPV-type distribu-

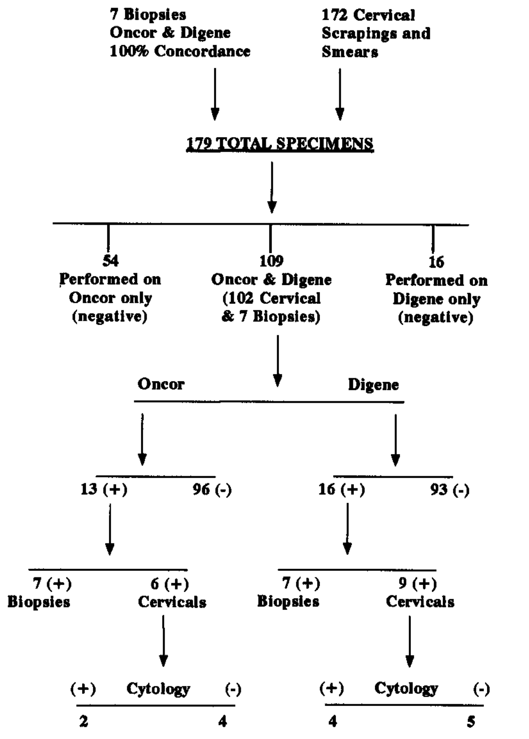

Fig. 1. Results of HPV nucleic-acid hybridization. Of a total of 179 specimens, 109 (102 cervical, 7 biopsies) were tested on both systems.

TABLE 1. HPV-type distribution in concordant specimens[a]

| | HPV type | | | | | | | Total |
|---|---|---|---|---|---|---|---|---|
| | 6 | 11 | 16 | 18 | 31 | 33 | 35 | no. |
| Oncor SB | 7 | 1 | 2 | 1 | 3 | 2 | 0 | 16 |
| Digene DB | 8 | | 3 | | | 5 | | 16 |
| | (50%) | | (18.8%) | | | (31.2%) | | |

[a] Results are given in number of positives.

tion and percentage for the concordant specimens without discrepancy. Two biopsy specimens were positive for >1 type: HPV types 6, 16, 33 and types 6, 16, respectively.

### Comparison With Cytology

One hundred forty-nine (83.2%) of the 169 specimens tested by SB and 114 (63.7%) specimens tested by DB were compared with the cytology results. There were 19/169 Papanicolaou smears graded SIL-L. There were no smears graded SIL-H or CIS in this patient population. The sensitivity, specificity, efficiency, and positive and negative predictive values of the Papanicolaou smears compared with SB were 33.3%, 89.5%, 87.3%, 11.8%, and 97.0% ($P = 0.085$). Compared with DB, they were 44.4%, 87.6%, 84.2%, 23.5%, and 94.9% ($P = 0.010$). Only the cytology results compared with DB were statistically significant. Based on this comparison, a Papanicolaou smear does not appear to predict HPV infection.

Two of the specimens positive only by DB for HPV types 6/11 and 16/18, respectively, also had positive cytology smears for SIL-L low/suggestive of HPV infection. An additional specimen positive only by DB for HPV types 31/33/35 was smear negative using the established criteria. There were no specimens positive by SB and negative by DB.

### Correlation With Biopsy Specimens

The histology results were available from cervical biopsy specimens collected on 21/172 patients participating in our study. Eight specimens were collected prior to enrollment in the study and 13 were collected after study completion. Ten of 21 patients were negative for HPV by hybridization and biopsy, while 1 biopsy specimen was positive both histologically and by SB (type 18) and DB (type 16/18). Eight of 21 patients had biopsy specimens with condylomatous changes and/or koilocytosis, but were negative for HPV by hybridization. One of these specimens, however, tested positive for HPV using a chemiluminescent molecular hybridization assay (Hybrid Capture System, Digene Diagnostics, Inc.) during a subsequent evaluation in the author's laboratory. Two of 21 patients had positive SB and DB tests for type 6/11, but were biopsy negative.

### Correlation of Risk Factors

Of the 7 potential risk factors for predicting HPV infection, only the number of lifetime partners was statistically significant in our study. Based on the logistic regression (Fig. 2) of the number of partners, the probability of acquiring HPV, with 1 partner considered the norm, is 0.017, increasing to 0.655 with 20 partners. The relative risk (Fig. 3) of acquiring HPV is 0.79 with 0 partners and 37.7 with 20 partners. For example, a person with 20 partners is 37 times more likely to become infected with HPV compared with a person with 1 partner. The relative odds risk (Fig. 4) of acquir-

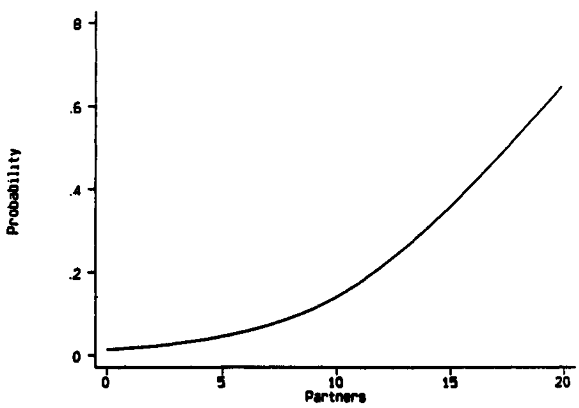

Fig. 2. Using logistic regression, the probability of acquiring HPV, with 1 partner as the norm being 0.017 and increasing to 0.655 with 20 partners.

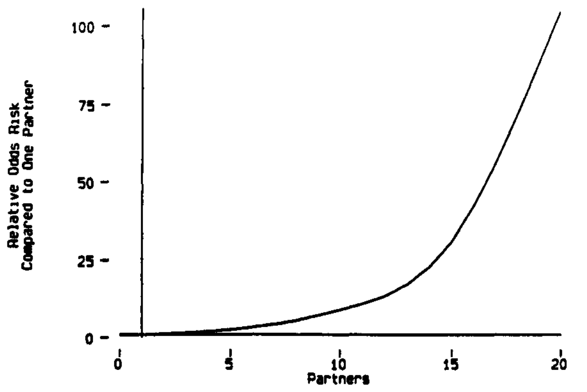

Fig. 4. Using 164 observations, the relative odds risk of acquiring HPV (106 times greater with 20 partners than with 1 partner).

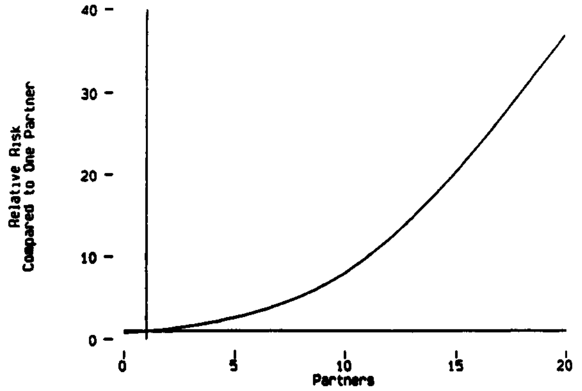

Fig. 3. Using 164 observations, the relative risk of acquiring HPV (0.79 with no partners and 37 times greater with 20 partners).

ing HPV is 106 times greater with 20 partners than with 1 partner.

### DISCUSSION

Infection with papillomavirus is a rapidly growing sexually transmitted disease in the United States today. Because of its association with cervical carcinoma, the ability to detect HPV in genital specimens has become an important issue in the management of patients with equivocal Papanicolaou smears. Until recently, cytology has been the only means of detecting potential HPV infection. Several methods have been developed for use in the clinical laboratory in order to detect the presence of HPV in infected tissue as well as the HPV type including SB, DB, in situ hybridization, and more recently hybrid capture.[14] In this evaluation, we compared the Oncor SB with the Digene DB technique to identify the presence of 7 of the most common HPV types (6, 11, 16, 18, 31, 33, and 35) in cervical specimens and penile biopsies collected from 179 patients. The SB method utilized biotinylated nucleic-acid RNA probes for the detection of the individual HPV types and the DB utilized $^{32}P$-labeled RNA probes for types 6/11, 16/18, and 31/33/35. The ViraType DB, rather than the ViraPap screening DB which utilizes a cocktail of the same probes, was chosen because the manufacturer stated that the ViraType was more sensitive. To date, reports comparing the results from these 2 kits have not been published. In addition, different risk factors and cytologic findings were assessed to further determine which group of individuals would most benefit from HPV testing.

SB hybridization is considered the gold standard for the identification of specific HPV types as well as the identification of new types and subtypes, although the SB procedure is technically challenging, labor intensive, and time-consuming, taking approximately 5 days to complete a batch of specimens. The Oncor procedure uses non-radiolabeled probes, which permits a long shelf life. Attention to detail and exact pipetting are required in order to obtain bands that are easily interpreted. The specimens collected in the Oncor transport medium must be stored at 4°C. The electrophoretic equipments' cost and technologist's time need to be considered when deciding to use SB vs. DB, although the

Oncor Probe Tech may be used for other molecular diagnostic tests such as gene rearrangement.

In contrast, the FDA-approved ViraType DB is relatively easy to perform and fast, being completed in 8 h, exclusive of autoradiography. The only equipment required are the manifolds and a vacuum pump. As a convenience, the specimens may be stored at room temperature for up to 2 weeks. A disadvantage of the DB method is the use of radiolabeled probes, with a shelf life of <2 weeks. As with the SB method, for cost effectiveness, specimens must be batched. According to the manufacturer's directions, bloody specimens may cause false negatives. False positives, on the other hand, may be caused by trapping of the probe by specimens containing significant protein and cellular debris. Unlike the SB, the ViraType DB only detects HPV groups 6/11, 16/18, and 31/33/35, rather than the individual types. However, the results may still provide the physician who wishes to test for HPV with information that can be used in patient management. An HPV-profile DB assay, which utilizes 2 probes (1 to identify low-risk HPV types 6, 11, 42, 43, and 44 and the other to detect intermediate and high-risk types 16, 18, 31, 33, 35, 45, 51, 52 and 56), is available for research use only (Digene Diagnostics, Inc.).

The specimens for HPV testing were collected with Christmas-tree cytobrushes at the same time the cytology smears were prepared in order to ensure that the cells were obtained from the same site. Each set of patient samples was combined, mixed, and reallocated to minimize sampling error. The collection of 3 specimens probably accounted for the high number of specimens with blood, i.e., 54/172 cervical specimens. Although Bartholoma et al.[15] utilized bloody specimens in their evaluation of Oncor $^{32}$P-labeled probes vs. the Digene ViraPap $^{32}$P-labeled probes for HPV screening, they extracted and precipitated the DNA first. This procedure, however, was not endorsed by Digene (personal communication). Retrospectively, 2 visibly bloody SB-positive specimens were tested by DB; both were positive. Due to the current recommendation by many cytopathologists, cytobrushes were used to collect the specimens. Other studies have utilized swabs rather than brushes, which might have accounted for a report of decreased sensitivity of the SB compared with the DB procedure.[16]

Of the 109 specimens tested in parallel, 97.3% were concordant for the presence and type of HPV. These findings are considerably higher than those of Burmer et al.,[17] Kiviat et al.,[18] and Bartholoma et al.,[15] who reported a concordance of 66% (102/154 specimens), 68% (62/91 specimens), and 78.7% (48/61 specimens), respectively. They also reported a higher number of positive specimens: 45/154 (29%),[19] 91/450 (20%),[20] and 31/61 (50.8%)[15] specimens vs. 13/179 (7.3%) positive specimens obtained in this study. The low percent of positive specimens in this study probably reflects the patient population selected. In contrast to other studies, the majority of specimens (146/179) were obtained from patients seen in a private OB/GYN practice. In concordance with the findings of Bartholoma et al.,[15] all biopsies tested were DB and SB positive.

Kiviat et al.[18] compared the ViraPap DB and SB using $^{32}$P-labeled probes. They reported sensitivity, specificity, and positive and negative predictive values of 90%, 94%, 74%, and 98%, respectively, which compares favorably with the results obtained in this study.

Six of the 9 positive cervical specimens were positive by both DB and SB in this study. Two of 3 specimens positive by DB only had corresponding positive Papanicolaou smears. Although reports have suggested that biotinylated probes are not as sensitive as the $^{32}$P-labeled probes in the DB procedure, they appear to have equal sensitivity and specificity in the SB procedure.[6,21] The remaining specimen that was positive by DB for types 31/33/35 and SB negative had a correpsonding negative Papanicolaou smear.

Only 11/21 (52.4%) of the hybridization and cervical biopsy histology results were concordant. Seven of the remaining histology results were positive, even though the HPV DNA results were negative. This may reflect a lack of sensitivity on the part of the DB and SB assays or perhaps a sampling variation. Due to the limited number of biopsies, we were unable to draw any conclusions.

Although SB is considered the gold standard based on its greater sensitivity and specificity,[21,22] when the results from the Oncor SB using $^{32}$P-labeled probes were compared with the ViraPap DB, it was reported to be less sensitive.[15,18] Perhaps this observation was due to a loss of DNA

during the extraction and transfer steps of the SB procedure, particularly if only a small quantity of DNA was present in the specimen. Whether our findings are consistent with Bartholoma et al.[15] and Kiviat et al.[18] or the 3 specimens that were positive by DB were, in fact, true positives cannot be determined with certainty. These specimens will be tested by a hybrid-capture chemiluminescent procedure (Digene Diagnostics, Inc.) (manuscript in preparation), a new 6-h procedure for detecting HPV DNA.

Bartholoma et al.[15] compared the results from cervical specimens using the Oncor SB $^{32}$P-labeled probes, and the ViraPap DB. Seventy-four percent (37/50 specimens) compared favorably. The discrepancies resulted from a lack of detection of HPV types 31/33/35 by SB. Eight specimens were positive by DB for types 31/33/35 and negative by the Oncor SB. One of these DB-positive specimens, which gave bands by SB, may have represented a cross-reaction, e.g., HPV type 16 shares a region of DNA homology with type 31.

In this study, the 3 discrepant specimens were deemed adequate during the microscopic-quality assessment procedure. The sampling variations were minimized or eliminated by combining the paired specimens and then distributing them in equal volumes for DB and SB testing. To avoid interobserver variation, each SB and DB test result was evaluated independently by 2 observers without prior clinical or Papanicolaou-smear information. A third individual was responsible forcoding the specimens prior to testing. There was 100% concordance of interpretations by the observers.

Weintraub et al.[23] compared the ViraType and cytology results and reported a concordance of 56% and a sensitivity and specificity of 48% and 77%, respectively. These results were similar to our findings, i.e., 84.2% concordance, 44.4% sensitivity, and 87.6% specificity, emphasizing the lack of predictability of the Papanicolaou smears for HPV infection. Although 19/169 Papanicolaou smears were reported as SIL-L, none was interpreted as SIL-H or CIS. Interestingly, 2 patients with previous normal Papanicolaou smears had smears showing squamous atypia and SIL-L. Both were positive for type 18 or 16/18. Infection with type 18 has been reported to occur in younger age groups (8–12 years old), cause a higher recurrence rate, and have the potential to rapidly progress to CIS within 1 year.[11]

Two of the 13 specimens (15.4%) were multiply infected with HPV. One biopsy was positive for HPV types 6, 16, and 33 and the second biopsy was positive for types 6 and 16. Dual infections are not unusual[14] and may represent exposure to multiple partners. Burmer et al.[17] reported a rate of 12% of multiple infections in their patient population.

Our study did not support the observation that an early onset of sexual relations increases the risk of HPV infection. However, only 42/172 patients enrolled in this study had their first sexual relations between 13 and 16 years of age. The only risk factor identified in this study was the number of sexual partners. Finding an association with the number of sexual partners is remarkable, considering the sample size.

In conclusion, the detection of HPV and identification of the specific type may have significant diagnostic and prognostic implications. To date, these viruses cannot be isolated in cell culture due to the requirement for cell differentiation in a productive or permissive infection, nor can serology be employed to provide a laboratory diagnosis. More sophisticated molecular diagnostic techniques have been developed utilizing DNA hybridization in order to detect this unique group of viruses.

Several testing formats are currently available for HPV DNA detection. The test method that is chosen should be easy to use, involve a reasonable turnaround time and minimal hands-on, employ a non-radioisotopic label for the probes, and provide excellent sensitivity, specificity, and predictive values, all at a reasonable cost. Although neither of the kits employed in this study fulfills all of these requirements, both methods have acceptable sensitivity, specificity, and predictive values when compared with cytology. Each kit has advantages and disadvantages. Both kits are designed for batching specimens due to the cost associated with the reagents, as well as the hands-on and turnaround time from start to finish of each run. With the implementation of stringent cost-containment programs throughout the country and decreases in laboratory staff in many hospitals, HPV testing may be considered in the following select cases: 1) history of another sexually transmitted disease, 2) equivocal Papanicolaou smear, 3) history of multiple sexual partners, and 4) immunocompromised status.

### ACKNOWLEDGMENTS

The authors express their thanks to James Dorsey, M.D., and Arthur Fetzer, M.D., for providing most of the specimens; Sandra Todd, Microbiology/Virology Division, for help in coding the specimens; Kathleen Moser, B.A., Research Department, for assistance in editing and preparation of the figures; and Lehigh Valley Hospital for the use of their facilities. A special thank you to Digene Diagnostics, Inc., and Oncor, Inc., for their technical support and for the supply of some of the kits for testing.

### REFERENCES

1. Andersen ES, Nielsen K, Larsen G: Laser conization: follow-up in patients with cervical intraepithelial neoplasia in the core margin. Gynecol Oncol 39:328–331, 1990.
2. Baggish MS: A comparison between laser excisional conization and laser vaporization for the treatment of cervical intraepithelial neoplasia. Am J Obstet Gynecol 155:39–44, 1986.
3. Whiteley PF, Olah KS: Treatment of cervical intraepithelial neoplasia: Experience with the low-voltage diathermy loop. Am J Obstet Gynecol 162:1272, 1990.
4. Richart RM, Wright TC: Controversies in the management of low grade cervical intraepithelial neoplasia. Cancer 71:1413–1421, 1993.
5. National Cancer Institute Workshop: The 1988 Bethesda System for reporting cervical/vaginal cytologic diagnoses. JAMA 262:931–934, 1989.
6. Wright TC, Richart RM: Role of human papilloma virus in the pathogenesis of genital tract warts on cancer. Gynecol Oncol 37:151–164, 1990.
7. Richart RM: Natural history of cervical intraepithelial neoplasia. Clin Obstet Gynecol 10:748, 1968.
8. Nasiell K, Roger U, Nasiell M: Behavior of mild cervical dysplasia during long-term follow-up. Obstet Gynecol 67:665, 1986.
9. zur Hausen H: Papilloma viruses in human cancer. Cancer 59:1692, 1987.
10. Lungu O, Sun WX, Felix J, Richart RM, Silverstein S, Wright TC: Relationship between human papilloma virus type and grade of cervical intraepithelial neoplasia. JAMA 267:2493–2496, 1992.
11. Lorincz AT, Reid R, Jenson AB, Greenberg MD, Lancaster W, Kurman RJ: Human papilloma virus infection of the cervix: Relative risk associations of 15 common anogenital types. Am J Obstet Gynecol 79:328–337, 1992.
12. Reid R, Lorincz AT: Should family physicians test for human papilloma virus infection? An affirmative view. J Fam Pract 32:183–188, 1991.
13. Lorincz AT, Schiffman MH, Jaffung WJ, Marlow J, Quinn AD, Temple GF: Temporal associations of human papilloma virus infection with cervical cytologic abnormalities. Am J Obstet Gynecol 162:644–651, 1990.
14. Brown DR, Bryan JT, Cramer H, Fife KH: Analysis of human papillomavirus types in exophytic condylomata by hybrid capture and Southern blot techniques. J Clin Microbiol 31:2667–2673, 1993.
15. Bartholoma NY, Adelson ND, Forbes BA: Evaluation of two commercially available nucleic acid hybridization assays for the detection and typing of human papillomavirus in clinical specimens. Am J Clin Pathol 95:21–29, 1991.
16. Yee C, Krisnan I, Baker CC, Schlegel R, Howley PM: Presence and expression of human papillomavirus sequences in human cervical carcinoma cell lines. Am J Pathol 119:361–366, 1985.
17. Burmer GC, Parker JD, Bates J, East K, Kulander BG: Comparative analysis of human papillomavirus detection by polymerase chain reaction and ViraPap/ViraType kits. Am J Clin Pathol 94:554–560, 1990.
18. Kiviat NB, Koutsky LA, Critchlow CW, et al.: Comparison of Southern transfer hybridization and dot filter hybridization for detection of cervical human papillomavirus infection with types 6, 11, 16, 18, 31, 33, and 35. Am J Clin Pathol 94:561–565, 1990.
19. Vessey MP: Epidemiology of cervical cancer: Role of hormonal factors, cigarette smoking and occupation. In Petro R, zur Hausen H (eds): Viral Etiology of Cervical Cancer. Cold Spring Harbor, NY: Cold Spring Harbor Laboratory Press, pp 29–43, 1986.
20. Kaplan IW: Condyloma acuminata. New Orleans Med Surg J 9:3, 1942.
21. Lorincz AT: Human papillomavirus testing. Diagn Clin Testing 27:28–37, 1989.
22. Choi YJ: Detection of human papillomavirus DNA on routine Papanicolaou smears by in situ hybridization with the use of biotinylated probes. Am J Clin Pathol 95:475–480, 1991.
23. Weintraub J, Mireille R, Seydoux J: The comparative test performance of dot filter hybridization (ViraType) and conventional morphologic analysis to detect human papillomavirus. Anat Pathol 97:46–57, 1992.
